# Retraction Note to: Engineering *Bacillus licheniformis* as a thermophilic platform for the production of l-lactic acid from lignocellulose-derived sugars

**DOI:** 10.1186/s13068-018-1086-z

**Published:** 2018-04-06

**Authors:** Chao Li, Zhongchao Gai, Kai Wang, Liping Jin

**Affiliations:** 1 Clinical and Translational Research Center, Shanghai First Maternity and Infant Hospital, Tongji University School of Medicine, Shanghai, 200092 People’s Republic of China; 2 State Key Laboratory of Microbial Metabolism, and School of Life Sciences & Biotechnology, Shanghai Jiao Tong University, Shanghai, 200240 People’s Republic of China

## Retraction to: Biotechnol Biofuels (2017) 10:235 10.1186/s13068-017-0920-z

The authors are retracting this article because the biological materials were used and the experiments were conducted without proper authorization from the laboratory where these data were obtained [1].
